# Effects of Resistance Training Experience on Bone Mineral Density and Stress Fractures in Female College Athletes: A Retrospective Cohort Study

**DOI:** 10.3390/sports13070227

**Published:** 2025-07-10

**Authors:** Tetsuro Kobayashi, Shotaro Seki, Mengrong Liu, Itaru Chiba, Takashi Oguro, Yosuke Makino, Yasunaga Kobayashi, Hiroyuki Matsumoto, Inkwan Hwang

**Affiliations:** 1 Department of Physical Education, Nippon Sport Science University, Tokyo 158-8508, Japan; hwang@nittai.ac.jp; 2 Department of Education, Ikuei University, Takasaki 370-0011, Japan; 3 Sport Training Center, Nippon Sport Science University, Tokyo 158-8508, Japan; seki.s@nittai.ac.jp (S.S.); t-oguro@nittai.ac.jp (T.O.); makino@nittai.ac.jp (Y.M.); kobayashi-yasunaga@nittai.ac.jp (Y.K.); h-matsumoto@nittai.ac.jp (H.M.); 4 Graduate School of Health and Sport Science, Nippon Sport Science University, Tokyo 158-8508, Japan; 24sda05@nittai.ac.jp; 5 Graduate School of Health Sciences, Hokkaido University, Sapporo 060-0808, Japan; chiba.itaru.n8@elms.hokudai.ac.jp

**Keywords:** athletes, bone mineral density, resistance training, sports, stress fracture

## Abstract

This study aimed to investigate the effects of resistance training (RT) experience on bone mineral density (BMD) and stress fractures (SFs) in female collegiate athletes. Overall, 492 female athletes from 16 competitive sports were included. Sports were categorized into four groups based on exercise load. Data on sports participation, RT experience, and SF history were obtained using a questionnaire. Total body and lumbar spine BMD were measured using dual-energy X-ray absorptiometry. Athletes with RT experience in both senior high school (ages 15–18) and university (ages 18–22), as well as those with experience from junior high school (ages 12–15) through university, had significantly higher BMD than those with no RT experience or RT experience only in senior high school (*p* < 0.05). Logistic regression analysis revealed that athletes with RT experience had significantly lower odds ratios for SFs compared to those with no RT experience. In the adjusted model that included sport type and university year, athletes with RT experience in junior high school, senior high school, and university had a significantly lower OR for SFs compared with no RT experience (OR = 0.06, 95% CI: 0.01–0.59, *p* = 0.016). No significant BMD differences were found between athletes with and without SFs (*p* > 0.05). The study findings suggest that initiating RT in junior high school may be associated with a reduced incidence of SFs during university.

## 1. Introduction

Stress fracture (SF) is a common sports injury defined as a partial or complete fracture of bone resulting from repeated stress that is lower than that required to fracture the bone in a single-loading situation [1]. The consequences of SF in athletes include pain, as well as the loss of training time and medical expenses [2]. The incidence of athletic SF is reportedly between 4.9 and 21.1% [3,4,5,6]. Teenagers may be at a higher risk of SF than young adults, with those aged 15–19 years representing the largest proportion of those afflicted in an athletic population (42.9%) [1]. Additionally, recurrence rates may be up to 21.5% in collegiate sports; thus, attention to risk factor management in this age group is indicated [4]. Risk factors for SFs include training load and early specialization in sports, low bone mineral density (BMD), female athletes, menstrual irregularities, hormones, low energy availability in male athletes, eating disorders, sleep and stress, biomechanical factors, prior SFs, high-magnitude loads that are introduced over short periods (e.g., sprinters), high number of load repetitions (e.g., distance running), and insufficient vitamin D and calcium intake [7,8,9,10]. In particular, female athletes have been reported to have approximately 1.5 times higher risk of SF than that in male athletes [11]. This can be attributed to the triad (low energy availability, amenorrhea, and osteoporosis) unique to female athletes that increases the risk of SF [12,13,14,15].

Resistance training (RT) has been discussed as a potential preventive method for SFs. Numerous studies have demonstrated that RT positively influences BMD [16,17,18,19]. RT can increase BMD within relatively short periods of less than 6 months [20,21,22]. In a study involving young females, Nickols-Richardson et al. [20] reported that a 5-month training regimen comprising concentric and eccentric RT performed three times a week led to significant increases in total body bone mass and site-specific BMD and bone mass. Mosti et al. [21] found significant increases in lumbar and femoral BMD following a 12-week RT program with squats performed at 85–90% of one-repetition maximum three times per week. Kobayashi et al. [22] reported a significant increase in total body BMD in female collegiate long-distance runners after 16 weeks of training twice per week involving squats and deadlifts. These findings suggest that the increase in BMD through RT can contribute to preventing SFs. Furthermore, it has been observed that 58% of high school athletes who developed bone stress injuries, ranging from periostitis and inflammation of the cortical bone itself to SFs over 2 years, did not engage in weight training, suggesting a protective effect [23]. However, the aforementioned studies did not clarify whether the risk of SF decreased with increased BMD. Moreover, the additional benefits of ongoing RT over multiple years or sporting seasons have not been explicitly investigated, and the optimal timing to load bone for injury prevention has not been well substantiated [7]. Accordingly, this study focuses on exploring how the timing of RT experience is associated with both BMD and the incidence of SFs in female collegiate athletes.

Therefore, the purpose of this study was to examine the effects of prior RT experience on BMD and SF incidence in female collegiate athletes. We hypothesized that athletes who initiated RT earlier would have higher BMD and lower SF rates during their college years.

## 2. Materials and Methods

### 2.1. Experimental Approach to the Problem

A retrospective cohort design was used to examine the effects of RT experience on BMD and the incidence of SFs in 492 female Japanese collegiate athletes. Participants were retrospectively categorized into exposure groups based on self-reported RT experience during junior high school, senior high school, and university. The primary outcome, SF incidence during university enrollment, was also assessed retrospectively through a physician-diagnosed injury history collected through a questionnaire. All data, including BMD measured by dual-energy X-ray absorptiometry (DEXA), were collected in August 2024.

### 2.2. Participants

Athletes who represented 16 competitive sports from a physical educational university (long-distance in track and field, *n* = 40; water polo, *n* = 35; lifesaving, *n* = 42; tennis, *n* = 25; soft tennis, *n* = 40; badminton, *n* = 27; handball, *n* = 51; basketball, *n* = 37; volleyball, *n* = 25; rhythmic gymnastics, *n* = 25; boxing, *n* = 14; fencing, *n* = 18; judo, *n* = 23; sprinting/jumping/throwing in track and field, *n* = 56; weightlifting, *n* = 28; trampoline, *n* = 6) participated in this study. Participants were required to be athletes who had continuously engaged in competitive sporting activities from junior high school through the time of the study. Participants were also required to be university students enrolled in their first to fourth year (ages 18–22). Athletes were excluded if they had a history of using hormone-based medications, such as low-dose estrogen preparations. A total of 530 athletes were initially recruited. All of them met the inclusion criterion of continuous sports participation since junior high school. However, five participants who engaged in non-competitive dance activities were excluded, as their sport was not classified as a competitive discipline in this study. In addition, 33 athletes were excluded due to the use of hormone-based medications. Consequently, data from 492 athletes were included in the final analysis. The physical characteristics of the participants are shown in Table 1.

The participants were informed of the benefits and risks of the investigation. If the individuals agreed to participate, they signed the institutionally approved informed consent document. The study was approved by the Ethics Review Committee of Nippon Sport Science University (023-H006) and conducted in accordance with the Declaration of Helsinki.

An a priori sample size calculation was conducted using G*Power version 3.1.9.7 (Heinrich Heine University Düsseldorf, Düsseldorf, Germany) to ensure adequate power for the primary analysis, which employed binary logistic regression. Based on a two-tailed test with a significance level of α = 0.05, a statistical power of 0.80, an assumed odds ratio of 0.30, a base event rate (stress fracture incidence) of 10%, and R^2^ = 0.10 for other predictors, the minimum required sample size was estimated to be 411 participants. Therefore, the actual sample size of 492 participants was deemed sufficient to detect statistically and clinically meaningful associations.

### 2.3. Procedures

For athletes, data on university years, sports participation, competition experience, RT experience, and history of SFs were collected through a questionnaire. The survey included the following content on RT experience. Participants were asked to check all the academic years during which they regularly performed RT (e.g., squats, deadlifts, bench presses, and cleans) using weights such as barbells or dumbbells at least once per week. If one or more academic years from junior high school (ages 12–15), senior high school (ages 15–18), or university (ages 18–22) were checked, the participant was classified as having engaged in RT during that period. The following contents were used in the survey to obtain the SF history. Only SFs diagnosed by a physician were included, and the participants were asked to specify the age and academic year at which the injury occurred, the affected body part, and the circumstances of the injury. In this study, we analyzed SFs that occurred during university enrollment.

The total body and lumbar spine L2–L4 BMD, fat mass, and lean mass were measured using DEXA scanning (iDXA; GE Medical Systems Lunar, Madison, WI, USA). DEXA scan preparation was as follows: the participants removed any pieces of metal from the body, including jewelry and dental appliances. Scans were performed wearing plain underwear and a common inspection gown (Figure S1). All the DEXA data were acquired by a single radiological technician.

Considering the effect of the characteristics of different sport types on BMD, the 16 competitive sports were classified into four categories based on previous studies [24] as follows: low-impact (long-distance in track and field), non-impact (lifesaving and water polo), multidirectional (tennis, soft tennis, badminton, handball, basketball, boxing, and rhythmic gymnastics), and high-impact (volleyball, fencing, judo, sprinting/jumping/throwing in track and field, weightlifting, and trampoline).

### 2.4. Statistical Analyses

All statistical analyses were performed using SPSS version 29.0 (IBM Corp., Armonk, NY, USA). The significance level was set at *p* < 0.05. A one-way analysis of variance was conducted to compare the BMD of athletes across different sport types and RT experiences. When significant effects were observed, Bonferroni post hoc tests were performed for multiple comparisons to identify significant differences between means. The comparison of BMD between groups with and without SFs was conducted using an independent *t*-test.

A binary logistic regression analysis was performed to predict the presence or absence of SFs during university enrollment. The dependent variable was the presence of SFs, coded as 1 for “present” and 0 for “absent.” The independent variable was RT experience, categorized as follows: (1) no RT experience; (2) RT experience in senior high school only; (3) RT experience in university only; (4) RT experience in both senior high school and university; and (5) RT experience in junior high school, senior high school, and university. The reference category was set as “no RT experience.” Additionally, three other categories (RT experience in junior high school only, RT experience in both junior and senior high school, and RT experience in both junior high school and university) were considered; however, no individuals met these criteria. Subsequently, an adjusted model was constructed by incorporating sport type and university year as additional independent variables as covariates. Sports types were categorized as follows: (1) low-impact, (2) non-impact, (3) multidirectional, and (4) high-impact sports. Low-impact sports were designated as the reference category, as they exhibited the highest incidence of stress fractures in the study cohort. University year was categorized as follows: (1) first-year, (2) second-year, (3) third-year, and (4) fourth-year, with first-year as the reference category. During the collinearity assessment, all Variance Inflation Factor (VIF) values were below 10, with a maximum VIF of 6.58 and a mean VIF of 3.73, indicating no serious multicollinearity issues. Effect sizes in the logistic regression models were reported as odds ratios (ORs) and 95% confidence intervals (CIs).

## 3. Results

### 3.1. SF Incidence

At the time of the survey, 53 athletes (10.8% of all participants) had experienced at least one SF during their university years, with 10 of them experiencing SF twice, resulting in 63 incidents. The anatomical locations and frequency of SFs were as follows: metatarsal (*n* = 11, 17.5%), femur (*n* = 11, 17.5%), tibia (*n* = 10, 15.9%), lumbar vertebrae (*n* = 9, 14.3%), medial malleolus (*n* = 6, 9.5%), navicular (*n* = 3, 4.8%), fibula (*n* = 2, 3.2%), pubis (*n* = 2, 3.2%), rib (*n* = 2, 3.2%), ulna (*n* = 2, 3.2%), sacrum (*n* = 2, 3.2%), calcaneus (*n* = 2, 3.2%), and carpus (*n* = 1, 1.6%). The number and percentage of athletes who experienced SFs by sport were as follows: long-distance in track and field (*n* = 18, 45.0%), sprinting/jumping/throwing in track and field (*n* = 9, 16.1%), rhythmic gymnastics (*n* = 4, 16.0%), badminton (*n* = 3, 11.1%), weightlifting (*n* = 3, 10.7%), basketball (*n* = 3, 8.1%), tennis (*n* = 2, 8.0%), handball (*n* = 4, 7.8%), water polo (*n* = 2, 5.7%), lifesaving (*n* = 2, 4.8%), judo (*n* = 1, 4.3%), volleyball (*n* = 1, 4.0%), soft tennis (*n* = 1, 2.5%), and boxing, trampoline, and fencing (*n* = 0, 0%). These findings highlight the variation in SF incidence across sports, with long-distance runners exhibiting the highest prevalence of SFs.

### 3.2. BMD Based on Each Sport Type and RT Experience

**Figure 1:**
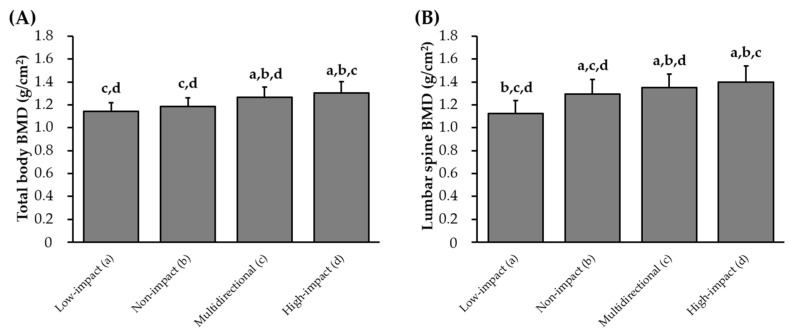
Comparison of total body (**A**) and lumbar spine (**B**) bone mineral density among sports types. Values are presented as mean ± standard deviation. BMD, bone mineral density; Low-impact, athletes participating in long-distance in track and field; Non-impact, athletes participating in lifesaving and water polo; Multidirectional, athletes participating in tennis, soft tennis, badminton, handball, basketball, boxing, and rhythmic gymnastics; High-impact, athletes participating in volleyball, fencing, judo, sprinting/jumping/throwing in track and field, weightlifting, and trampoline; ANOVA, analysis of variance. ^a^ *p* < 0.05 vs. low-impact; ^b^ *p* < 0.05 vs. non-impact; ^c^ *p* < 0.05 vs. multidirectional; ^d^ *p* < 0.05 vs. high-impact.

**Figure 2:**
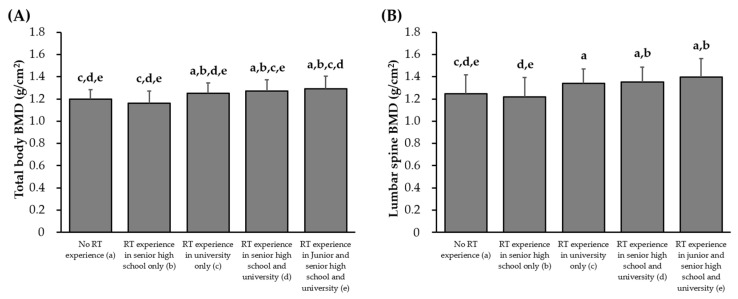
Comparison of total body (**A**) and lumbar spine (**B**) bone mineral density among resistance training experience. Values are presented as mean ± standard deviation. BMD, bone mineral density; RT, resistance training. ^a^ *p* < 0.05 vs. no RT experience; ^b^ *p* < 0.05 vs. RT experience in senior high school only; ^c^ *p* < 0.05 vs. RT experience in university only; ^d^ *p* < 0.05 vs. RT experience in senior high school and university; ^e^ *p* < 0.05 vs. RT experience in junior and senior high school and university.

The BMDs of athletes based on each sport type and RT experience are shown in Figure 1 and Figure 2. Additional details on physical characteristics by sport are provided in Supplementary Material (Table S1). Low- and non-impact athletes had significantly lower total body and lumbar spine BMD than that of multidirectional and high-impact athletes (*p* < 0.05, respectively). Athletes with RT experience during senior high school and university, as well as junior high school, senior high school, and university, had significantly higher total body and lumbar spine BMD than those of females with no RT experience and RT experience only during senior high school.

### 3.3. Effects of RT Experience on SFs

**Figure 3:**
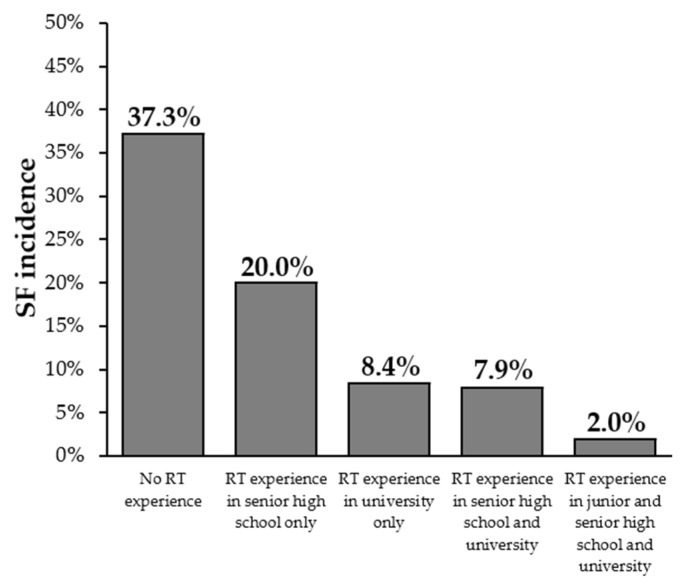
The incidence rates of stress fractures during university enrollment based on resistance training experience. SF, stress fracture; RT, resistance training.

The incidence rates of SFs during university enrollment based on RT experience are shown in Figure 3. The highest incidence of SFs was observed in athletes with no RT experience (37.3%), whereas the lowest incidence was found in those who had RT experience in junior high school, senior high school, and university (2.0%).

In the binary logistic regression analysis, athletes with RT experience in university only; both senior high school and university; and junior high school, senior high school, and university had significantly lower ORs compared with that of athletes with no RT experience (OR = 0.16, 95% CI: 0.08–0.35, *p* < 0.001; OR = 0.16, 95% CI: 0.07–0.35, *p* < 0.001; OR = 0.04, 95% CI: 0.01–0.28, *p* = 0.002, respectively). Furthermore, in the adjusted model that included sport type and university grade, athletes with RT experience in junior high school, senior high school, and university had a significantly lower OR compared with that of athletes with no RT experience (OR = 0.06, 95% CI: 0.01–0.59, *p* = 0.016) (Table 2).

### 3.4. Comparison of BMD Between Athletes with and Without SFs During College

No significant differences were observed in total body and lumbar spine BMD between athletes without and with SFs during their college years (1.258 ± 0.097 vs. 1.234 ± 0.131 g/cm^2^, *p* = 0.188; 1.342 ± 0.139 vs. 1.309 ± 0.195 g/cm^2^, *p* = 0.227, respectively).

## 4. Discussion

This is the first study to investigate the effects of RT experience on BMD and SF risk during college in female athletes. The findings of this study demonstrated that athletes with RT experience in university only, in both senior high school and university, or junior high school, senior high school, and university had significantly lower ORs for SFs compared with the ORs of those with no RT experience. Furthermore, in the model adjusted for sport type and university year, athletes with RT experience in junior high school, senior high school, and university still had a significantly lower OR for SFs than those with no RT experience. These findings support our hypothesis that the earlier initiation of RT is associated with lower SF incidence during the college years and suggest that initiating such training as early as junior high school may be associated with a reduced incidence of SFs in female collegiate athletes.

The efficacy of RT in preventing acute and chronic (overuse) sports injuries has been supported in previous studies [25]. However, there is limited research investigating its effects on SFs. Nussbaum et al. [23] analyzed factors related to overuse injuries in adolescent athletes across various sports. Their findings revealed that 58% of athletes who developed bone stress injuries had no prior weight training experience. Moreover, athletes with bone stress injuries engaged in RT at an average frequency of 1.03 sessions/week, significantly lower than the 2.12 sessions/week observed in the control group. The results of the present study demonstrated that athletes with RT experience limited to university or both senior high school and university did not differ significantly in SF risk compared with the risk of those without RT experience after adjusting for sport type and university year. However, athletes with RT experience from junior high school through university exhibited a significantly lower risk of SF compared with the risk of those without any RT experience. While no prior studies have specifically examined the relationship between the timing of RT experience and SF risk, these findings suggest the importance of initiating RT during early developmental stages, such as junior high school, to reduce the risk of SF during university years. Conversely, Tenforde et al. [6] reported no significant differences in the training experience (e.g., weightlifting and plyometrics) between female adolescent runners with and without SF. This suggests that the impact of RT experience on SF risk may vary depending on the sport. However, it is important to note that Tenforde et al. did not investigate RT experience retrospectively by academic stages, limiting comparability with the present study.

Many studies have demonstrated that RT enhances BMD in various populations, including young adults, older adults, and athletes [16,17,18,19]. Moreover, its positive effects have been reported even in relatively short training durations, such as within 6 months [18,19,20]. The mechanism by which RT increases BMD is thought to involve mechanical loading on bones, which stimulates bone formation [26]. Exercises such as squats and deadlifts, which impose high mechanical loads on the skeletal system, are particularly effective in promoting bone formation responses [27]. The anabolic effects of RT operate through mechanical and hormonal pathways, with acute increases in hormones such as growth hormone, testosterone, and insulin-like growth factor-1 observed after intense exercise sessions [28]. In this study, athletes with RT experience during both senior high school and university, or from junior high school through university, had significantly higher BMD compared with the BMD of athletes with no RT experience, supporting the findings of previous studies [16,17,18,19,20,21,22]. Although the present findings indicate that athletes with RT experience have higher BMD, it is important to recognize that BMD can be affected by various factors, including the type of sport [24]. Therefore, future prospective studies are needed to determine whether RT experience has beneficial effects on BMD within each specific sport discipline.

In the present study, no significant differences in total body or lumbar spine BMD were observed between athletes who sustained SFs during their university years and those who did not, suggesting that other factors may mediate the protective effects of RT against SFs. Existing studies on the relationship between low BMD and SFs have produced inconsistent results [3,29,30,31,32]. For example, Nose-Ogura et al. [15] conducted a prospective study on female athletes in their teens and twenties and reported that low BMD was associated with SFs in teenage athletes but not in athletes in their twenties. They attributed this difference to greater muscle mass in the older athletes, suggesting that muscle mass may influence SF risk. Furthermore, Bennell et al. [3] reported that runners with smaller calf circumferences are more prone to SFs and proposed that increasing muscle mass might reduce SF risk. Bennell et al. [3] also suggested that muscles act as a cushion, reducing the impact on bones during ground contact, thereby lowering the risk of SF [33]. The present study included university athletes aged 18–22, which might have contributed to the lack of an observed association between low BMD and SFs. Although BMD accounts for approximately 70% of bone strength [34], the remaining 30% is influenced by bone quality, including bone geometry, collagen properties, and the microarchitecture of trabecular and cortical bone [35]. Some studies have reported associations between bone geometry and SFs [36,37], highlighting the need for analyses that consider both bone quality and BMD. Additionally, comprehensive RT incorporating plyometric exercises such as jumping and landing during adolescence has been shown to enhance biomechanics, improve functional capacity, and reduce sports-related injuries [38]. Therefore, athletes who start RT during junior high school may reduce their risk of SF during university through early improvements in biomechanics and functional performance. Future studies should evaluate factors beyond BMD, such as bone quality, muscle strength, and functional capacity, to elucidate the mechanisms by which RT may prevent SFs.

### Limitations

This study has several limitations. First, it relied on self-reported data regarding the incidence of SFs, which introduces the possibility of recall bias. Although only physician-diagnosed SFs were included, participants might not have accurately recalled the timing, site, or circumstances of the injury. Second, although this study employed a retrospective cohort design, BMD could not be measured at the time SFs occurred. This limitation may have contributed to the lack of an observed association between BMD and SFs. Third, training variables (e.g., type, intensity, volume, and frequency) were not clearly distinguished, nor was it possible to identify the specific skeletal sites that were protected against SFs. In addition, regression analysis using RT experience as the dependent variable could not be conducted due to insufficient detail in the dataset. Future research should investigate these variables in greater detail to clarify RT parameters that effectively reduce the risk of SF. Fourth, the inability to analyze data by sport type is another limitation. SF-prone sites and the bones subjected to the greatest mechanical loads vary by sport. Addressing this limitation will require large-scale investigations stratified by sport type, as well as interventional studies on RT. Finally, participants were limited to university students aged 18–22. Consequently, many young female athletes of a similar age who did not attend university were excluded from the analysis. This might have introduced selection bias and limited the generalizability of the findings to the broader population of young female athletes, particularly those pursuing non-university athletic careers, such as employment-based or professional sports pathways.

## 5. Conclusions

To the best of our knowledge, no prior studies have examined the relationships among RT experience, BMD, and SFs in female collegiate athletes. This study demonstrated that athletes with RT experience during university, both senior high school and university, or from junior high school through university, had a significantly lower risk of SF than athletes with no RT experience. Additionally, even after adjusting for sport type and university year, athletes who underwent RT in junior high school and continued through university had a significantly lower risk of SF than those with no prior RT experience. These findings suggest that initiating RT as early as junior high school may be associated with a reduced incidence of SFs during university years. The findings of this study support the efficacy of RT for young athletes [39,40]. While planning training programs, athletes, coaches, physicians, and parents can incorporate this knowledge and initiate RT in athletes during junior high school to help prevent SFs during university.

## Figures and Tables

**Table 1:** The physical characteristics of the participants.

	All Participants (*n* = 492)
Age (y)	20.2 ± 1.4
Height (cm)	161.0 ± 6.0
Weight (kg)	57.8 ± 7.6
BMI (kg/m^2^)	22.3 ± 2.5
%Fat (%)	14.2 ± 3.9
Fat mass (kg)	41.3 ± 4.6
Fat free mass (kg)	24.2 ± 4.3

Values are presented as mean ± standard deviation. BMI, body mass index; SD, standard deviation.

**Table 2:** Binary logistic analysis of effects of resistance training experience, sport type, and university year on stress fractures.

Categories		*n*	Non-Stress Fractures		Stress Fractures		Unadjusted Model		Model Adjusted for Sport Type		Model Adjusted for Sport Type and University Year	
							OR (95%CI)	*p*	OR (95%CI)	*p*	OR (95%CI)	*p*
RT experience												
	No experience	51	32	(62.7%)	19	(37.3%)	1.00 (reference)		1.00 (reference)		1.00 (reference)	
	Senior high school only	10	8	(80.0%)	2	(20.0%)	0.44 (0.09–2.32)	0.336	0.57 (0.10–3.30)	0.532	0.50 (0.08–3.33)	0.477
	University only	191	175	(91.6%)	16	(8.4%)	0.16 (0.08–0.35)	**<0.001**	0.50 (0.16–1.56)	0.230	0.35 (0.11–1.13)	0.080
	Senior high school and university	189	174	(92.1%)	15	(7.9%)	0.16 (0.07–0.35)	**<0.001**	0.50 (0.15–1.64)	0.249	0.42 (0.13–1.41)	0.161
	Junior and senior high school and university	51	50	(98.0%)	1	(2.0%)	0.04 (0.01–0.28)	**0.002**	0.10 (0.01–0.91)	**0.041**	0.06 (0.01–0.59)	**0.016**
Sport type												
	Low-impact	40	22	(55.0%)	18	(45.0%)			1.00 (reference)		1.00 (reference)	
	Non-impact	77	73	(94.8%)	4	(5.2%)			0.13 (0.03–0.59)	**0.009**	0.15 (0.03–0.70)	**0.016**
	Multidirectional	219	202	(92.2%)	17	(7.8%)			0.17 (0.06–0.54)	**0.002**	0.25 (0.08–0.79)	**0.018**
	High-impact	156	142	(91.0%)	14	(9.0%)			0.24 (0.07–0.84)	**0.026**	0.45 (0.12–1.64)	0.227
University year												
	First-year	103	101	(98.1%)	2	(1.9%)					1.00 (reference)	
	Second-year	118	112	(94.9%)	6	(5.1%)					2.69 (0.52–14.07)	0.240
	Third-year	88	79	(89.8%)	9	(10.2%)					6.25 (1.27–30.78)	**0.024**
	Fourth-year	183	147	(80.3%)	36	(19.7%)					12.97 (2.94–57.29)	**<0.001**

OR, odds ratio; CI, confidence interval; RT, resistance training; Low-impact, athletes participating in long-distance in track and field; Non-impact, athletes participating in lifesaving and water polo; Multidirectional, athletes participating in tennis, soft tennis, badminton, handball, basketball, boxing, and rhythmic gymnastics; High-impact, athletes participating in volleyball, fencing, judo, sprinting/jumping/throwing in track and field, weightlifting, and trampoline.

## Data Availability

Data sets generated during the current study are available from the corresponding author on reasonable request.

## Supplementary Materials

The following supporting information can be downloaded at: https://www.mdpi.com/article/10.3390/sports13070227/s1. Figure S1: DEXA scan image; Table S1: Physical characteristics by sport.

## Abbreviations

The following abbreviations are used in this manuscript:

BMD	Bone mineral density
CI	Confidence interval
DEXA	Dual-energy X-ray absorptiometry
OR	Odds ratio
SF	Stress fracture
RT	Resistance training
