# Supplementary material for: Effects of Resistance Training Experience on Bone Mineral Density and Stress Fractures in Female College Athletes: A Retrospective Cohort Study
Source: Sports (Basel). 2025 Jul 10;13(7):227. doi: 10.3390/sports13070227 (PMC12298017; doi:10.3390/sports13070227)
Supplement: Supplementary file 1 [file sports-13-00227-s001.zip › Table S1.pdf]

Table S2. Physical characteristics of each sport

Sport	n	Height (cm)	Weight (kg)	BMI (kg/m²)	%Fat (%)	Fat mass (kg)	Fat free mass (kg)	Total body BMD (g/cm^2)	Lumbar spine BMD (g/cm^2)
Long-distance in track and field	40	158.9 ± 5.3	48.1 ± 3.8	19.1 ± 1.4	19.8 ± 3.0	9.7 ± 1.8	37.0 ± 2.6	1.144 ± 0.072	1.124 ± 0.112
Water polo	35	162.0 ± 5.2	59.5 ± 7.2	22.6 ± 2.2	24.0 ± 4.1	14.4 ± 3.6	42.6 ± 4.5	1.189 ± 0.081	1.306 ± 0.129
Lifesaving	42	160.4 ± 5.2	57.3 ± 5.5	22.3 ± 1.6	25.6 ± 3.6	14.8 ± 3.1	40.2 ± 3.2	1.185 ± 0.072	1.284 ± 0.130
Tennis	25	160.2 ± 5.9	55.7 ± 5.2	21.7 ± 1.8	25.6 ± 3.3	14.3 ± 2.6	39.0 ± 3.6	1.244 ± 0.087	1.337 ± 0.095
Soft tennis	40	158.4 ± 4.6	56.7 ± 5.0	22.6 ± 1.6	27.2 ± 3.6	15.5 ± 2.9	38.8 ± 3.2	1.234 ± 0.067	1.314 ± 0.122
Badminton	27	161.0 ± 5.2	58.5 ± 5.8	22.5 ± 1.9	24.4 ± 3.2	14.3 ± 2.9	41.7 ± 3.8	1.299 ± 0.055	1.372 ± 0.114
Handbal	51	161.8 ± 5.5	60.0 ± 6.5	22.9 ± 2.0	24.5 ± 4.3	14.9 ± 3.8	42.6 ± 3.6	1.282 ± 0.088	1.365 ± 0.115
Basketball	37	166.6 ± 7.1	61.5 ± 7.5	22.1 ± 1.8	22.1 ± 4.0	13.7 ± 3.7	45.1 ± 4.7	1.316 ± 0.092	1.337 ± 0.123
Volleyball	25	166.6 ± 6.8	62.2 ± 5.7	22.4 ± 1.6	25.6 ± 3.3	15.9 ± 2.7	43.5 ± 4.1	1.314 ± 0.072	1.407 ± 0.109
Rhythmic gymnastics	25	159.2 ± 4.5	52.2 ± 4.9	20.6 ± 1.6	22.9 ± 2.8	12.1 ± 2.2	37.9 ± 3.4	1.221 ± 0.099	1.410 ± 0.113
Boxing	14	157.4 ± 4.5	54.1 ± 4.4	21.8 ± 1.4	23.7 ± 3.7	12.9 ± 2.8	38.9 ± 2.2	1.230 ± 0.078	1.304 ± 0.108
Fencing	18	160.1 ± 5.9	55.7 ± 8.0	21.6 ± 1.9	24.9 ± 3.0	13.8 ± 2.5	39.5 ± 6.0	1.222 ± 0.084	1.245 ± 0.107
Judo	23	159.3 ± 4.6	63.3 ± 6.1	24.9 ± 2.3	25.6 ± 4.3	16.3 ± 3.3	44.3 ± 4.5	1.366 ± 0.091	1.492 ± 0.110
Sprinting/jumping/throwing in track and field	56	162.2 ± 4.8	59.2 ± 9.5	22.5 ± 3.0	22.7 ± 5.1	13.8 ± 5.2	43.0 ± 4.8	1.288 ± 0.096	1.368 ± 0.128
Weightlifting	28	156.9 ± 5.6	62.5 ± 8.5	25.4 ± 2.8	26.1 ± 5.0	16.6 ± 5.1	43.3 ± 4.3	1.331 ± 0.089	1.471 ± 0.136
Trampoline	6	157.9 ± 4.9	53.1 ± 5.9	21.3 ± 2.2	25.1 ± 3.9	13.5 ± 3.1	37.3 ± 3.5	1.275 ± 0.119	1.459 ± 0.160

Values are presented as mean ± standard deviation. BMI, body mass index; SD, standard deviation.
