# Supplementary figures and images for: Effects of Resistance Training Experience on Bone Mineral Density and Stress Fractures in Female College Athletes: A Retrospective Cohort Study
Source: Sports (Basel). 2025 Jul 10;13(7):227. doi: 10.3390/sports13070227 (PMC12298017; doi:10.3390/sports13070227)

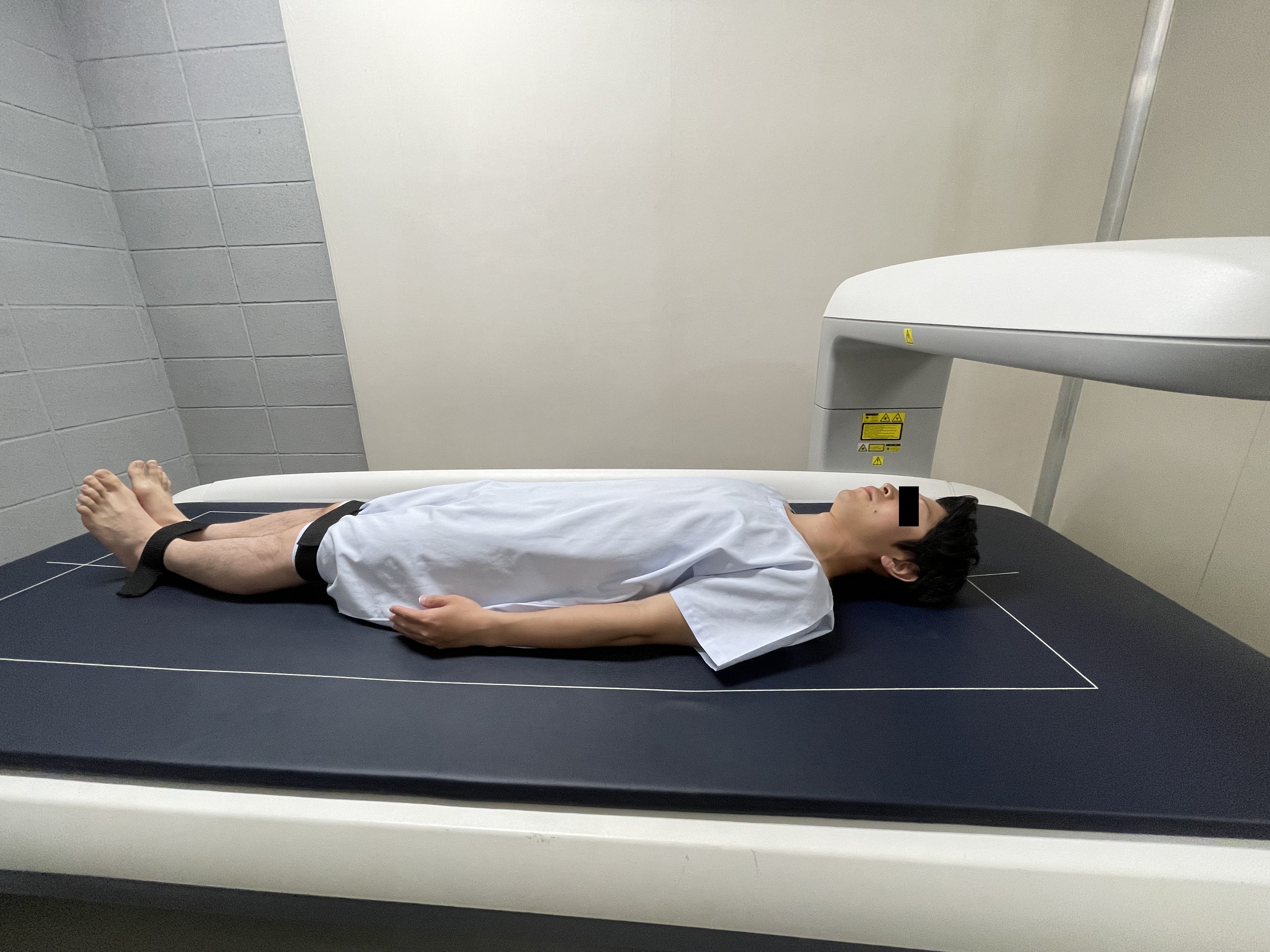

Supplement: Supplementary file 1 [file sports-13-00227-s001.zip › Figure S1.jpg]
